# Animal Models in Studies of Cardiotoxicity Side Effects from Antiblastic Drugs in Patients and Occupational Exposed Workers

**DOI:** 10.1155/2014/240642

**Published:** 2014-02-19

**Authors:** Monica Lamberti, Giancarlo Giovane, Elpidio M. Garzillo, Franca Avino, Antonia Feola, Stefania Porto, Vincenzo Tombolini, Marina Di Domenico

**Affiliations:** ^1^Department of Experimental Medicine, Section of Hygiene, Occupational Medicine and Forensic Medicine, Area of Occupational Medicine, Second University of Naples, Via L. De Crecchio 7, 80138 Naples, Italy; ^2^National Institute for the Study and Treatment of Cancer, Foundation “G. Pascale”, Via Mariano Semmola, 80131 Napoli, Italy; ^3^Department of Biochemistry, Biophysics and General Pathology, Second University of Naples, Via L. De Crecchio 7, 80138 Naples, Italy; ^4^Department of Radiology, Oncology and Pathological Anatomy Sciences, University of Rome, “La Sapienza”, Piazzale Aldo Moro 5, 00185 Rome, Italy; ^5^Spencer-Lorillard Foundation, University of Rome, “La Sapienza”, Piazzale Aldo Moro 5, 00185 Rome, Italy; ^6^Sbarro Institute for Cancer Research and Molecular Medicine, Center for Biotechnology, Building Suite 431, 1900 North 12th Street, Philadelphia, PA 19122, USA

## Abstract

Cardiotoxicity is an important side effect of cytotoxic drugs and may be a risk factor of long-term morbidity for both patients during therapy and also for staff exposed during the phases of manipulation of antiblastic drugs. The mechanism of cardiotoxicity studied in vitro and in vivo essentially concerns the formation of free radicals leading to oxidative stress, with apoptosis of cardiac cells or immunologic reactions, but other mechanisms may play a role in antiblastic-induced cardiotoxicity. Actually, some new cytotoxic drugs like trastuzumab and cyclopentenyl cytosine show cardiotoxic effects. In this report we discuss the different mechanisms of cardiotoxicity induced by antiblastic drugs assessed using animal models.

## 1. Introduction 

Many anticancer drugs have, as side effect, the risk of severe cardiotoxicity by a cumulative, dose-dependent toxicity for both patients during therapy and also for healthcare workers during the phases of manipulation of antiblastic drugs (Table 1). In fact, several scientific studies have shown that in exposed workers the presence of several cardiotoxic drugs like doxorubicin, epirubicin, cyclophosphamide, and 5-fluourouracil was often identified in urine [1, 2]. Cardiotoxicity effects include small changes in blood pressure as well as arrhythmias and cardiomyopathy [3]. Mechanisms of cardiotoxicity by antiblastic drugs comprise cellular damage, with the formation of free oxygen radicals and the induction of immunogenic reactions with the presence of antigen presenting cells in the heart [4]. Early and late onset cardiac effects are reported; the first effect can be acute, subacute, or chronically progressive [5]. Acute or subacute cardiotoxicity effects of antiblastic drugs are rare; they occur during or immediately following infusion and are usually transient (e.g., electrocardiographic abnormalities such as nonspecific ST-T changes and QT prolongation, pericarditis-myocarditis syndrome, and ventricular dysfunction with congestive heart failure) [6]. The late effect generally starts within one year after the beginning of antiblastic therapy with chronic cardiac abnormalities and can progress to overt cardiac disease. However a sudden atrial fibrillation was observed at the third week of chemotherapy administration in patients with myotonic dystrophy [7]. The clinical symptoms may include all signs of cardiomyopathy with electrophysiologic changes, decrease of left ventricular function, changes in exercise-stress capacity, and overt signs of congestive heart failure [8]. During administration of taxoids, as paclitaxel, combined or with cisplatin, various cardiac disturbances, like brady- and tachyarrhythmias, atrioventricular and bundle branch blocks, and cardiac ischemia were reported [9]. Evidence of hypotension is also described, probably correlated to hypersensitivity reaction. A combination of doxorubicin and paclitaxel administration in rats is correlated to an increase of myocardial necrosis compared with those treated with DOX alone [10].

5-Fluorouracil (5-FU) has direct toxic effects on vascular endothelium that involves endothelial nitric oxide synthase and leads to coronary spasms and endothelium-independent vasoconstriction via protein kinase C [11, 12]. Cardiotoxicity effects of 5-FU include cardiac arrhythmias, silent myocardial ischemia, angina, congestive heart failure, and even sudden death [13]. Various neoadjuvant chemoradiation therapies of squamous cell carcinoma are reported in the literature. They consisted of a combination between radiotherapy treatments and mitomycin-C and 5-fluorouracil [14]. A recent large meta-analysis shows that notwithstanding ongoing improvements in chemotherapy treatments, anthracyclines still represent a considerable risk of cardiotoxicity [15].

Other cytotoxic drugs that have been reported to be cardiotoxic are capecitabine, mitoxantrone, cisplatin, and newer drugs, like the monoclonal antibody trastuzumab or melphalan, fludarabine, mitomycin, busulfan, mechlorethamine and dacarbazine [16]. New generation of tyrosine kinase inhibitors (TKIs), like sorafenib and sunitinib, are associated with direct cardiotoxicity [17]. Since the antiblastic-induced cardiotoxicity is generally irreversible, it is crucial to detect the myocardial injury at its earliest possible stage; for this reason several experimental studies on cell cultures or animal models have been carried out.

Results on toxic effects of antiblastic drugs in various species were found extremely variable. Not only does the LD50 vary from species to species [18] but the qualitative character of the pharmacodynamic action of the drug also is equally varied [19]; therefore we can only partially compare precisely the dose and the toxic effects of antiblastic drugs between the model and the human animal model.

The major point of attack may be either the central nervous system or the heart. The rabbit is a representative animal showing cardiac responses [20], while in the dog [21] effects on central nervous system are the main response; rhesus monkey produces mixed-type responses [22]. However, despite the large number of investigations made, the results obtained in animal models are still hard to be translated to humans; therefore there is a critical need for continued translational research and animal studies to improve our understanding of the molecular mechanisms that underlie the cardiac dysfunction of antiblastic drugs.

## 2. Mechanisms of Cardiotoxicity

### 2.1. Role of Oxidative Stress

Antiblastic drugs, as most of xenobiotics, are generally metabolized through the NADPH-cytochrome P450 system in order to increase their solubility in urine. In particular, doxorubicin could be substrate of several oxidoreductases like NADH-dehydrogenase of mitochondrial complex I and various cytoplasmic oxidoreductases, including xanthine oxidase. The oxidoreductive reaction starts with a single electron transfer from NADPH to doxorubicin forming a semiquinone radical that is complexed with iron ion in a ferrous form; this complex is responsible for the oxygen reduction, thus producing a superoxide ion [8]. The superoxide free radicals generated in mitochondria have cardiolipin as a preferential target. Cardiolipin is a major phospholipid component of the inner mitochondrial membrane and is required for the activity of respiratory chain. It is rich in polyunsaturated fatty acids and is particularly susceptible to peroxidative injury [23]; furthermore, evidence has been reported showing a strong affinity of doxorubicin for cardiolipin [24]. The drug-phospholipid complex formation leads to an inhibition of mitochondrial enzymes involved in oxidative phosphorylation. The mitochondrial membrane damage can also generate the inactivation of key transporters involved in ion homeostasis. Thus, the well-known cardiotoxicity of anthracycline could simply explain considering the fact that the cardiac tissue is rich in mitochondria. However, other factors are involved in anthracycline cardiotoxicity among which the relative lower amount of antioxidant defenses of heart compared with other tissues. It should be also considered that when the levels of free radicals increase, the apoptotic cascade is activated by cytochrome c being released from the damaged mitochondria, thus triggering apoptosis. Vásquez-Vivar et al. [25] have shown that doxorubicin binds to the reductase domain of endothelial nitric oxide synthase causing an increase in superoxide and a decrease in nitric oxide formation.

### 2.2. Role of Cytokines

Anthracyclines produce a drug-related systemic inflammation which has been found to be mediated by interleukins [26]. In particular, interleukin-1beta (IL-1beta) has been implicated in this mechanism. Doxorubicin induces a systemic increase in IL-1beta and other inflammatory cytokines, chemokines, and growth factors including TNF-alpha, IL-6, CXCL1/Gro-alpha, CCL2/MCP-1, granulocyte colony stimulating factor, and CXCL10/IP-10. Studies on mice deficient in IL-1 receptor demonstrate that IL-1 signaling plays a role in the increase of IL-6 and GCSF induced by doxorubicin. The IL-1beta release required the expression of caspase-1, NLRP3, and the adaptor protein ASC indicating that inflammation is mediated by the NLRP3 inflammasome. The molecular mechanisms by which anthracyclines trigger IL-1beta release are not completely understood; however the undesirable consequences of anthracyclines due to their inflammatory activity that complicate chemotherapy may be reduced by suppressing the actions of IL-1beta. It has been also showed that the administration of anthracyclines to mice having cancer stimulates the secretion of tumor necrosis factor alpha (TNF-alpha) in neoplastic tissue [27]. The antineoplastic effects of anthracyclines could be partially due to a local immune response that involves several distinct subsets of T lymphocytes and dendritic cells. However, the blockage of the TNF-alpha/TNF receptor system did not influence the antineoplastic effects of doxorubicin against MCA205 fibrosarcomas growing in C57BL/6 mice, F244 sarcomas developing in 129/Sv hosts, and H2N100 mammary carcinomas in BALB/c mice. These findings show that, in contrast to other cytokines, TNF-alpha is not required to elicit anticancer immune responses. Aluise and coworkers [28] demonstrate that doxorubicin oxidizes plasma APOA1 that, in turn, enhances macrophage TNF-alpha release contributing to a possible TNF-alpha-mediated toxicity. Furthermore they produced evidence that reducing agent 2-mercaptoethane sulfonate blocks this mechanism suggesting that this antioxidant could reduce systemic side effects of doxorubicin.

Doxorubicin has been also showed to be a potent inducer of apoptosis in both cardiomyocytes H9c2 and osteosarcoma tumor cells U2OS; however, caspase activation and kinetics take place with significant differences between the two cell lines [29]. In fact, apoptosis is accompanied by relevant changes in levels of TNF-alpha receptor in H9c2 cardiomyocytes but not in U2OS cells. Moreover, treatment with exogenous TNF-alpha strongly increases the apoptotic effect of doxorubicin in H9c2 cardiomyocytes but not in U2OS cells. The function of TNF receptors I and II is differently affected by doxorubicin which induces in H9c2 cells an increase in the death domain-containing TNFR-1 protein levels and a decrease in the survival domain-containing TNFR-2 protein levels. These findings demonstrate a balance between proapoptotic and antiapoptotic signaling pathways in the cardiomyocyte survival after TNF stimulation showing a relevant role of TNF-alpha receptor-mediated signaling in cardiotoxicity induced by anthracyclines.

### 2.3. Calcium Homeostasis

Another aspect to be considered is the effect of anthracyclines on the role played by mitochondria in calcium homeostasis [30]. In fact the drug-induced malfunction of transporters involved in ion homeostasis can lead to a loss of mitochondrial calcium loading capacity which is observed in several in vitro and in vivo models [31, 32]. Alterations in calcium transport can lead to tissue damage impairing the cardiac contraction. In vitro experiments demonstrate that doxorubicin treatment produces an irreversible decrease in mitochondrial calcium loading capacity. Moreover, anthracyclines could stimulate “in vitro” the release of calcium from isolated sarcoplasmic reticulum. In rodent models a decrease of calcium loading capacity together with alterations in cardiac mitochondrial function has been observed [33]. Verapamil, a calcium blocking agent, shows a protective effect against doxorubicin cardiotoxicity [34]. The antagonizing effect could be due to the ability of verapamil to inhibit the intracellular calcium overload. Contradictory results, however, arise from experiments showing an increase of cardiotoxicity when doxorubicin was given in combination with verapamil [35]. A possible explanation for this discrepancy could be due to the capacity of verapamil to inhibit the function of P-glycoprotein and therefore may increase intracellular cytotoxic drug concentrations. Other authors found that the additive cardiotoxicity of verapamil was due to its selective inhibition of cardiac actin gene [36], an effect which was also demonstrated with doxorubicin alone. Tagliaferri and coworkers [37] found side effects on heart electric conductance following infusion of high dose verapamil incorporated into cytotoxic chemotherapy. Several symptoms like premature ventricular beats, and mild and transient hypotension were observed. Hypokalemia was also detected probably as a consequence of transient activation of the renin-angiotensin system.

### 2.4. Metabolite Theory

To overcome the cardiotoxic effect of anthracyclines the use of antioxidants have been suggested [38, 39], however antioxidants has proven to be useful in delaying or preventing chronic cardiotoxicity in rodents [40] but not in dogs [41] or sheep [42]. For patients, contradictory results have been reported showing positive [43, 44] or no [45] effect. Taking into account these discrepancies, a new hypothesis has been made on the evidence that chronic cardiomyopathy develops after conversion of doxorubicin to the corresponding secondary alcohol metabolite doxorubicinol [46]. This metabolite is formed after the reduction of carbonyl group on the C-13 side chain; the reaction is probably mediated by cytoplasmic oxidoreductases [47]. The secondary alcohol metabolite production is suggested by several lines of evidence: (i) in rodents, after anthracycline treatment, a decline of cardiac function usually is observed when alcohol metabolite reachs its maximum levels in the heart [48]; (ii) overexpression of human carbonyl reductase in transgenic mice heart produces an accelerated development of cardiomyopathy [49]; (iii) modified anthracyclines with resistance to reduction of carbonyl moiety produce a less severe chronic cardiotoxicity in rats [50]. Due to their chemical structures, secondary alcohol metabolites are considerably less effective than their parent drugs at producing oxygen radicals, probably for their reduced affinity for quinone reductases [51]. However, secondary alcohol metabolites are several times more potent at inactivating membrane ATPases [52] and cytoplasmic aconitase/iron regulatory protein 1 [53]. The evidence that secondary alcohol metabolites can be involved in chronic cardiomyopathy suggested the hypothesis that the clinical use of anthracyclines could be improved by reducing their conversion to secondary alcohol. This goal could be reached in at least two ways: (i) a chemical modification of drugs to produce less alcohol metabolites and (ii) a development of inhibitors of reductases which are responsible for transformation of ketone/aldehyde moiety to alcohol. Obviously the investigations on the inhibitors have to consider possible differences in specificity and affinity between the reductases of humans and those of laboratory animals used to verify the protective efficacy of these inhibitors.

Recently [54] an effect of glutamine against oxidative damage due to doxorubicin has been reported. The free radicals produced by doxorubicin result in a decrease of glutathione (GSH) and a depletion of superoxide dismutase in cardiac muscle [55]. It seems that glutamine has a protective role in the myocardial cell by upregulating GSH and also by inducing the synthesis of heat shock protein 72 [56]. This protein is known to protect the myocardium against hypoxic/ischemic injury. Furthermore, the induction of heat shock protein 27 has been shown to be protective against cardiac injury induced by doxorubicin [57]. Glutamine also appears to be a potent inducer of myocardial HSP 72 in an in vivo rat model. Recently evidence has been produced indicating that glutamine can preserve the level of high-energy phosphate in myocardial tissue and prevent the stress-dependent accumulation of lactate, including ischemia/reperfusion injury [58].

It is now well assessed that anthracyclines possess the ability to bind covalently to DNA; the bind is strictly dependent on the availability of formaldehyde [59]. In fact, formaldehyde supplies the carbon required for the N–C–N linkage necessary for the adduct formation with DNA. The resulting adduct is further stabilized by the formation of hydrogen bond with the complementary strand of DNA to crosslink the DNA duplex resulting in stabilization of the local region of DNA.

Doxorubicin is also known to intercalate itself into the DNA, with the inhibition of both DNA and RNA polymerase, thus blocking DNA replication and RNA transcription [60]. Recently it has been reported that doxorubicin is capable of intercalating with not only nuclear DNA but also mitochondrial DNA [61].

### 2.5. Tyrosine Kinase Inhibitors

Recent years have seen significant progress in cancer therapy through the development of “targeted therapies”, in particular those using TKIs directed against certain tyrosine kinases whose abnormal activity triggers cancer development and progression through cell proliferation and neoangiogenesis. Multikinase inhibitors have been widely used alone and in combination with other drugs in cancer therapies for different tumor types [62, 63]. Unfortunately, due to their large spectra of action, these inhibitors are also associated with toxicity to the heart [64, 65]. For example, sunitinib inhibits a number of growth factor receptors regulating both tumor cell proliferation/survival and tumor angiogenesis including vascular endothelial growth factor receptors, platelet-derived growth factor receptors *α* and *β*, c-Kit, FLT3, CSF1R, and RET [66]. However, care should be taken when cardiotoxicity in humans and animal models is compared. In fact it has been reported [67] that while the TKIs pazopanib, sunitinib and sorafenib, showed cardiotoxic effects in humans, studies in animal model failed to show cardiac toxicities for all of these TKIs. TKIs can be divided into two general classes: (i) humanized monoclonal antibodies directed against the tyrosine kinase receptor or their ligands and (ii) small molecules interacting with kinases inhibiting their activity. The use of both classes of TKIs revealed a relatively high rate of adverse cardiac events in the clinic, with systolic dysfunction and resultant heart failure as one of the most common and important side effects. TKIs are frequently used for the treatment of renal-cell carcinoma, gastrointestinal stromal tumors, and other tumor types in which these drugs are still under investigation. It seems that TKIs have as target AMPK which is a critical kinase controlling the balance between ATP and AMP levels [66]. Following conditions of energy stress, AMPK may act as a metabolic switch, increasing energy generation and inhibiting anabolic pathways. Studies on animals treated with sunitinib suggest that together with a potential misregulation in AMPK signaling a possible role is played by mitochondrial dysfunction leading to alterations in cardiac energy homeostasis. Most probably sunitinib induces a cardiac dysfunction that could be dependent on the simultaneous inhibition of multiple signaling pathways all of which are necessary for the preservation of cardiac function and which could play a pivotal role in the increased cardiac stress such as hypertension [68].

## 3. Other Cardiotoxicity Mechanisms

### 3.1. Taxoids

Paclitaxel is formulated in a cremophor EL vehicle to enhance the drug solubility and it is suggested that the vehicle and not the cytotoxic drug itself is responsible for the cardiac disturbances. However, the cardiac rhythm disturbances are not reported with use of other drugs containing cremophor EL such as cyclosporine [69, 70]. The possible mechanism by which cremophor EL would cause cardiotoxicity is massive histamine release. Indeed, stimulation of histamine receptors in cardiac tissue in animal studies has resulted in conduction disturbances and arrhythmias. An alternative explanation for paclitaxel induced cardiotoxicity could be the induction of cardiac muscle damage by affecting subcellular organelles. Enhanced cardiac toxicity has been found in combined therapy of paclitaxel and doxorubicin. A similar effect has been shown for epirubicin. Docetaxel shows no increase in cardiac toxicity when combined with doxorubicin.

### 3.2. Cyclophosphamide and Ifosfamide

High dose cyclophosphamide is used in transplant regimens and is associated with acute cardiotoxicity such as cardiac decompensation as well as fatal cardiomyopathy. Acute reversible decrease in systolic function has been described. Ifosfamide cardiotoxicity is reported in only a single study. The pathogenesis is not fully understood but an increase in free oxygen radicals seems to play a role in oxazaphosphorine induced cardiotoxicity. This increase would be mediated by elevated intracellular levels of the actual cytotoxic metabolite phosphoramide mustard [71].

### 3.3. Cisplatin

Several factors have been suggested to be involved like vascular damage, alterations in platelet aggregation, and hypomagnesemia [72]. In experiments on animal platelets, cisplatin was able to trigger platelet aggregation and/or enhance thromboxane formation by platelets. Activation of an arachidonic pathway in platelets by cisplatin seemed to be involved [73].

### 3.4. Trastuzumab

Trastuzumab is a monoclonal antibody directed against the HER2 receptor protein on breast cancer cells and it has been used alone or in combination with other chemotherapeutic agents. Cardiac toxicity associated to trastuzumab seems to be similar with the congestive heart failure observed with anthracycline therapy [74].

## 4. Concluding Remarks

In the context of modern cancer chemotherapeutics, cancer survivors are living longer and being exposed to potential comorbidities related to noncancer side effects of such treatments as cardiotoxicity. These same toxic effects can also be detected in healthcare worker exposed during the manipulation of chemotherapy because several studies have identified the presence of drugs such as doxorubicin, epirubicin, cyclophosphamide, and 5-fluorouracil in these subjects. These side effects can be cause of severe morbidity and even mortality, so knowledge about their incidence and mechanism is important. The authors have reevaluated in the different articles available in the scientific literature the possible causes of cardiotoxicity due to administration of antiblastic drugs by using animal models. In fact, animal models have historically been unable to predict human response to drugs and this is the basis for their widespread use in human toxicity testing. The mechanisms of action described in the literature are different, such as, the oxidative stress for doxorubicin and misregulation in AMPK signaling by TKI. These results disclose a new scenario for prevention of heart complications.

We are now, in fact, able to identify specific early biomarker of chemotherapy cardiotoxicity, discovered on animal models, and to develop supportive therapies to reduce or eliminate the appearance of these side effects in humans.

## Figures and Tables

**Table 1:** Effects of antiblastic drugs on heart.

Drugs	CHF*	ECG change	Bradycardia	Ischemia	Arrythmias	Myo/pericarditis	Hypotension	Hypertension
Anthracyclines	++	++			+	+		
Trastuzumab	++							
5-FU	+	+		++	+			
Cisplatin		++		++				++
Bleomycin		+				+		
Cyclophosphamide	+++	++				++		
Methotrexate		+					+	
Doxorubicin	+	+						+
Taxoids (paclitaxel)		++	+	+	++		+	
Mitoxantrone					+		+	
TKIs (sorafenib, sunitinib, etc.)	+++							++

*CHF: congestive heart failure.
